# Pathways linking school bullying and psychotic experiences: Multiple mediation analysis in Chinese adolescents and young adults

**DOI:** 10.3389/fpsyt.2022.1007348

**Published:** 2022-10-28

**Authors:** Lu Hua Chen, Timothea Toulopoulou

**Affiliations:** ^1^Department of Rehabilitation Sciences (Neuroscience and Neurological Rehabilitation), Faculty of Health and Social Sciences, The Hong Kong Polytechnic University, Kowloon, Hong Kong SAR, China; ^2^Mental Health Research Centre, The Hong Kong Polytechnic University, Kowloon, Hong Kong SAR, China; ^3^Department of Psychology, Faculty of Social Sciences, The University of Hong Kong, Kowloon, Hong Kong SAR, China; ^4^Department of Psychology, Bilkent University, Ankara, Turkey; ^5^Department of Basic and Clinical Neuroscience, Institute of Psychiatry Psychology and Neuroscience at King's College London, London, United Kingdom

**Keywords:** school bullying, psychotic experiences, self-esteem, personality traits, interpretation bias

## Abstract

It is found that people with psychotic experiences have a 4-fold increased risk of developing a psychotic disorder later in life. Indeed, accumulating evidence has suggested that the association between school bullying and psychotic experiences works linearly. Previous studies are mainly carried out in a Western context, and only seldomly do studies address whether the association exists in the Chinese population and the related psychological and cognitive mechanisms. Therefore, we carried out the current study to address this gap in the literature focusing on the lifelong school bullying experiences of Chinese adolescents and young adults. We examined them in relation to psychotic experiences while assessing the mediating role of self-esteem, the personality trait of neuroticism, and a cognitive bias in thinking called interpretation bias. We found that multiple victimizations were quite common in Hong Kong secondary schools. In addition to a significant association between school bullying and psychotic experiences, we found partial mediating effects of proposed psychological and cognitive mediators in constructed multiple mediation models utilizing bootstrapping approach. Specifically, bullying quantity reflecting the number of victimizations, had its association with psychotic experiences partially mediated by the personality trait of neuroticism. In contrast, bullying duration reflecting the lasting of victimization was associated with psychotic experiences partially mediated by the personality trait of neuroticism and interpretation bias. Our findings enhance our knowledge of mechanisms underpinning the psychosis spectrum development and have implications for school-based intervention programs targeting bullying victims.

## Introduction

School bullying is an ever-growing problem with high prevalence all over the world. It is estimated that about 10–27% of school students are bullied on a regular basis worldwide (1). Generally, school bullying is defined as perpetrators' repeated imbalance of power to hurt, tease, force, abuse, and intimidate their victims. Those repetitive and aggressive behaviors can happen in and outside school by verbal or physical means (2). In Hong Kong, bullying has become particularly worrying in schools and has increased in recent decades. In 2004, around 18.3% of secondary school students were found to be victims of bullying (3). In 2010, the number was raised to 70.8%, according to Hong Kong Family Welfare Society's report (the most recent to date) based on data from 8 secondary schools, which was higher than findings in Western countries (53–68%) (4). During the COVID-19 pandemic, school bullying at primary and secondary schools is high, even if face-to-face classes are suspended (5). The impact of school bullying is long-term and profound. Accumulating evidence has indicated its association with an increased likelihood of psychosis across the continuum of symptom severity from subclinical psychotic experiences to clinical psychotic disorders later in life (6–8). Unlike in Western countries involving using shooting weapons during school violence, physical, verbal, and relational bullying are more common in Hong Kong. In the context of Chinese culture, the education at school of Hong Kong generally emphasizes on students' academic performance instead of their psychological and mental health and most school bullying is regarded as common conflicts among peers. At present, research on the impact of school bullying is dominated by Western countries. Local research on the consequences of school bullying is of little attention and quite sparse.

Psychotic experiences are expressions of the psychosis phenotypes at levels below clinical manifestation (9, 10), which consist of perceptual aberrations (11), magical thinking (12), hallucinations, and delusions (13). The experiences are common in adolescents and young adults, with an 8% prevalence in the general population. Approximately 10–20% of people with psychotic experiences will develop a psychotic disorder later in life, with a 4-fold increased risk of disease development (13, 14). Therefore, investigating the subclinical population with psychotic experiences will enhance our knowledge of those risk factors which contribute to psychosis as well as facilitate early intervention for those susceptible individuals.

Both cross-sectional and prospective studies support an association between school bullying and psychotic experiences (15–19), with bullied children showing a 2-fold increased risk of developing psychotic experiences in adolescence or later in adulthood compared to non-bullied controls. In a meta-analysis conducted by van Dam et al. (20), the victims of bullying have a 2.3-fold increased risk of developing psychotic experiences after adjustment of age, gender, and adverse life events. Furthermore, school bullying has been found to take effect in a linear fashion, with a more severe and frequent bullying history predicting a higher risk of developing psychotic experiences which are longer-lasting (16, 21). However, those findings are mainly derived from Western populations, and few studies to date have addressed whether the association exists in the Chinese population.

Although the underlying psychological mechanisms are unknown, it is assumed that several indirect pathways may be involved in the association between school bullying and psychotic experiences. One of the hypothesized indirect pathways is *via* self-esteem, as evidence has suggested the involvement of emotion processing which demonstrates a significant relationship between school bullying and lower self-esteem (22, 23). It has been found that the higher the frequency of being bullied, the lower the self-esteem of the victims (24). Findings from Egan et al. (25) have suggested that lower self-esteem may play an essential role in creating a vicious cycle that perpetuates and solidifies a child's status as a victim of school bullying. Additionally, lower self-esteem has been found to increase the susceptibility to the onset of auditory hallucinations and persecutory delusions (26, 27). Moreover, paranoid thinking has been reported to be negatively correlated with self-esteem (28). It is thought that victims of peer bullying may develop negative beliefs about themselves, lowering their self-esteem and ultimately leading to referential ideas, paranoid thinking, and misinterpretation of normal stimuli (29).

The second hypothesized indirect pathway is *via* personality traits, especially the neuroticism dimension characterized by emotional instability, proneness to anxiety, negative states, and a tendency to be vulnerable to stress (30). The neuroticism trait has been found to be involved in the individual's differences in emotional responses and susceptibility to mental health problems. Significant association has been reported between neuroticism trait and personality disorders, including paranoid, avoidant, schizotypal, and schizoid personality disorders (31, 32). Furthermore, evidence has suggested a significant association between neuroticism trait and psychosis, with increased neuroticism trait associated with increased disease risk (33) and decreased quality of life (34). As school bullying is defined to occur in a social context determined by the individual characteristics of the participants, personality traits have been investigated, and the findings suggested that victims scored higher on the neuroticism dimension following the violent interaction (35, 36). Thus, victims with higher neuroticism traits are assumed to be more vulnerable to environmental stress caused by school bullying, which may result in psychotic experiences. In addition to self-esteem and the neuroticism trait, the third hypothesized indirect pathway is *via* cognitive biases, which associations with school bullying have been implied by a recent meta-analysis (37) and have been previously studied in psychosis (29, 38, 39). For example, Buchy et al. (38) have demonstrated that cognitive biases are associated with higher scores on a schizotypy scale in non-clinical participants. Interpretation bias, one of the main cognitive biases, reflects the tendency to interpret ambiguous or neutral stimuli as threatening and has been suggested to play an important role in developing psychotic experiences in bullying victims (40). Recently, studies have focused on using Cognitive Bias Modification Therapy to modify negative interpretation bias to a positive one. Turner et al. (41) successfully showed beneficial changes in interpretation bias in people experiencing social anxiety following psychotic episodes by using this therapy. Due to the great potential of translating findings into clinical intervention, the linking mechanism induced by interpretation bias is thus warranted for our further exploration.

Therefore, in light of the literature findings, the current study's overall aim was to investigate the potential pathways linking school bullying and psychotic experiences as defined by the Community Assessment of Psychic Experiences (CAPE) in a population of Chinese adolescents and young adults. We speculated on the potential mediating roles of self-esteem, the trait of neuroticism, and interpretation bias using a multiple mediation model. We hypothesized that lower self-esteem, higher neuroticism trait/lower stability trait, and less positive/more negative interpretation bias would compose indirect pathways underlying the relationship between school bullying and subclinical psychotic experiences.

## Materials and methods

### Participants

Using a convenience sample, we conducted a cross-sectional study with participants recruited at Hong Kong public universities and education institutions. The Human Research Ethics Committee (HREC) of the University of Hong Kong approved the current study. Written informed consent was obtained from each participant ≥18 years old. Additional consent from parents was obtained for participants <18 years after explaining the nature of the study in detail. The inclusion criteria of the participants were Chinese adolescents and young adults aged between 15 and 24 years old, who had completed their secondary school, were able to read Chinese, and without any medical record of mental illness. Sample size was calculated using the Monte Carlo Power Analysis for Indirect Effects developed by Schoemann et al. (42). A minimum of 116 participants was required to detect a correlation of 0.30 (43) (standard deviation of 1.00) among the proposed 5 variables with 80% power. All participants were recruited by email circulars, telephone, flyers, and posters, with interested participants arranged for a face-to-face experiment with our research staff.

### Measures

#### Questionnaires

##### Retrospective bullying questionnaire (RBQ)

RBQ was employed to assess the participant's bullying history during secondary school by peers, which has shown good test-retest reliability (44). Six types of victimization were assessed, including: 2 items for physical bullying (1. Have you been physically bullied at secondary school by being beaten/hit/kicked?, 2. Have you been physically bullied at secondary school by being stolen from?), 2 items for verbal bullying (1. Have you been physically bullied at secondary school by nicknames?, 2. Have you been physically bullied at secondary school by being threatened?), and 2 items for relational bullying (1. Have you been indirectly bullied in secondary school by lies/gossip?, 2. Have you been indirectly bullied in secondary school by being excluded?). The internal consistency analysis indicates that RBQ has a reliability with Cronbach's Alpha coefficient of 0.96 in the current study. Bullying intensity variables were constructed using the following two parameters: (1) quantity, based on participant's experience with any subtypes of peer victimization encountered; and (2) duration, based on participant's responses on a 5-point scale which reflects how long bullying attacks lasted (“never,” “several days,” “weeks,” “months,” and “a year or more”). Each response was then used to compose a bullying duration variable by coding 0 = “never,” 1 = “several days to weeks,” 2 = “months to a year or more”. The two composed variables (bullying quantity and bullying duration) were then summarized to reflect a general bullying intensity index used for data analysis.

##### Community assessment of psychic experiences (CAPE)

CAPE, a self-report questionnaire comprising 42 items, was used to assess the general population's subclinical expression of psychotic experiences (45). The scale measures three dimensions of psychotic experiences, including positive, negative, and depressive dimensions on a 4-point scale (“never,” “sometimes,” “often,” and “nearly always”). The positive dimension (20 items) measures delusional thoughts, beliefs, and hallucinations, while the negative dimension (14 items) measures anhedonia, diminished affective responses, and social withdrawal. The depressive dimension (8 items) measures depressive thoughts. Previous studies has demonstrated good reliability and validity of the CAPE questionnaire (46–48). In the current study, the internal consistency analysis indicates that CAPE has a reliability with Cronbach's Alpha coefficient of 0.93. The overall scores were calculated by summing scores on the frequency scale divided by the total number of the completed questions.

##### Rosenberg self-esteem scale (RSES)

RSES is a 10-item self-report questionnaire examining participants' perceptions of self-worth (49). Five items are written in a positive manner, while the other five are negatively worded and reversely scored. Participants were asked to imply the strength of their agreement with each item on a 4-point scale (“strongly disagree,” “disagree,” “agree,” and “strongly agree”). The internal consistency analysis indicates that RSES has a reliability with Cronbach's Alpha coefficient of 0.85 in the current study. The overall score was calculated by summing the scores for 10 items, with higher scores indicating higher self-esteem.

##### Eysenck personality questionnaire (short form revised version) (EPQ-S)

EPQ–S, a revised version of the Eysenck Personality Questionnaire (50) was used to evaluate the personality traits in two dimensions, namely extraversion/introversion and neuroticism/stability, which have been more commonly found to be associated with psychotic disorders (33). Extraversion/introversion measures an individual's sociability and impulsivity, whereas neuroticism/stability measures emotional instability and reactiveness, such as being anxious, depressive, and having excessively emotional traits. Participants were asked to respond to each question by choosing either “yes” or “no” based on 24 items. A score of 1 was given if the answer was consistent with extraversion or neuroticism traits. Otherwise, a score of 0 was given. The internal consistency analysis indicates that EPQ-S has a reliability with Cronbach's Alpha coefficient of 0.84 in the current study. The scores on two different dimensions were summed separately. Only scores on the neuroticism dimension were used for data analysis, with higher scores indicating more neuroticism traits and less stability traits.

#### Computer-based cognitive task

##### Ambiguous situation task (AST)

The ambiguous situation task was employed to measure interpretation bias using the E-Prime 2.0 software. This task comprises two consecutive sessions. For the first session, participants were shown 10 ambiguous social situations. For each social situation, the last word of the last sentence on the computer screen missed a letter, and the participants were asked to fill it out. To ensure participants had paid attention to the social situation, they were immediately asked to answer a question about the situation by “yes” or “no”. For the second session, participants were presented with possible positive and negative interpretations of the social situations to the test passages of the first session. Four sentences (1 = “not similar at all,” 2 = “not very similar,” 3 = “similar,” and 4 = “very similar”) were presented with each statement, and participants were instructed to rate how similar it was to the social situation in the first session (51). The similarity ratings' mean scores were calculated separately for positive and negative scenarios. The differences between mean scores of positive and negative scenarios, which implied the interpretation bias, were used for data analysis, with a higher index indicating more positive interpretation bias and a lower index indicating more negative interpretation bias.

### Procedures

The face-to-face experiment lasting around 45–60 min was conducted by research staff at the laboratory of the Department of Psychology, Faculty of Social Sciences, the University of Hong Kong. After collecting the general demographic information of the participants, they were required to complete four questionnaires in Cantonese including RBQ, CAPE, RSES, and EPQ-S, which were administered *via* the online platform LimeSurvey (https://www.limesurvey.org/). Participants filled in those online surveys using the computer in our laboratory. Subsequently, AST, the computer-based cognitive assessment, was done by each participant with instruction from the research staff. The data from online surveys and AST were reviewed for completeness by the research staff once the experiment was completed.

### Statistical analysis

Statistical analyses were carried out using Statistical Package of Social Science (SPSS) version 23. Logistic regression analyses were carried out to compare bullying quantity differences between genders. Multiple linear regressions were conducted to examine the specific associations of each mediator (self-esteem, neuroticism trait, and interpretation bias) with bullying intensity (quantity and duration) during secondary school by peers, and psychotic experiences, separately. Finally, we used multiple mediation analyses which utilized the Preacher and Hayes's bootstrapping approach implemented in SPSS *via* Syntax to test the proposed mediating pathways (52). The latter is a non-parametric procedure to create an empirical approximation of the sampling distribution and generates a confidence interval (CI) to test the indirect effect related to the mediation model. All mediation analyses were adjusted by potential confounding variables of age, gender, and education levels with 10,000 bootstrapping resamples applied. The *p* < 0.05 was regarded as statistically significant in the current study.

## Results

### Participant characteristics

Two hundred and sixty twoo participants were finally recruited, including 243 singleton participants and 19 single twin participants from the TwinsscanChina project [a twin-based study for psychotic experiences in Hong Kong (47, 53)]. As shown in Table 1, there were 95 males (36.3%) and 167 females (63.7%). 92.7% of the participants received bachelor's degree education, while 7.3% received secondary school education. The most common type of bullying was “Received nickname” of verbal bullying (36.3%) and “Being gossiped and told lies” of social bullying (36.3%). 141 (53.8%) participants reported having secondary school bullying experiences covering physical, verbal, and social bullying. Among the 141 participants being bullied, 55 suffered from 1 subtype of bullying (39%), 36 participants suffered from 2 subtypes of bullying (25.5%), 29 participants suffered from 3 subtypes of bullying (20.6%), 11 participants suffered from 4 subtypes of bullying (7.8%), 7 participants suffered from 5 subtypes of bullying (5.0%), and 3 participants suffered from 6 subtypes of bullying (2.1%). Using logistic regression by controlling confounder effects of age and education levels, male participants were found to experience more physical bullying of “being beaten/kicked/hit” (OR = 5.12, *p* = 0.007) and verbal bullying of “being given nickname” (OR = 2.41, *p* = 0.001) compared to female participants (Table 2).

**Table 1:** Characteristics of all participants.

	**All participants (*n* = 262)**
Age, years (mean ± SD)	19.73 ± 1.68
**Gender**
Male (*n*, %)	95 (36.3%)
Female (*n*, %)	167 (63.7%)
**Education level**
Secondary school (*n*, %)	19 (7.3%)
Bachelor degree (*n*, %)	243 (92.7%)
**Secondary school bullying experiences**
Yes (*n*, %)	141 (53.8%)
No (*n*, %)	121 (46.2%)
**Subtypes of secondary school bullying experiences**
**Physical bullying**
Being beaten/kicked/hit	16 (6.1%)
Things were being stolen (*n*, %)	17 (6.5%)
**Verbal bullying**
Received nick name (*n*, %)	95 (36.3%)
Being verbally threaten	26 (9.9%)
**Social bullying**
Being gossiped and told lies (*n*, %)	95 (36.3%)
Social exclusion (*n*, %)	63 (24.0%)
RSES score (mean ± SD) (*n*, %)	28.52 ± 5.35
EPQ-S (neuroticism/stability) score (mean ± SD)(*n*, %)	6.88 ± 3.47
AST score (mean ± SD)	0.44 ± 0.52
CAPE score (mean ± SD)	1.31 ± 0.96

RSES, rosenberg self-esteem scale; EPQ-S, eysenck personality questionnaire (short form revised version); AST, ambiguous situation task; CAPE, community assessment of psychic experiences.

**Table 2:** Comparison of high school bullying experiences by gender.

**Bullying subtypes**	**All participants (*****n*** = **262)**			
	**Male (*n* = 95)**	**Female (*n* = 167)**	P*	OR**(95% CI)
**Physical bullying**
Being beaten/kicked/hit (*n*, %)	11 (11.6%)	4 (2.4%)	**0.007**	**5.12 (1.57–16.67)**
Things were being stolen (*n*, %)	9 (9.5%)	8 (4.8%)	0.102	2.30 (0.85–6.25)
**Verbal bullying**
Being given nickname (*n*, %)	47 (49.5%)	48 (28.7%)	**0.001**	**2.41 (1.42–4.09)**
Being verbally threatened (*n*, %)	12 (12.6)	14 (8.4%)	0.224	1.67 (0.73–3.81)
**Social bullying**
Being gossiped and told lies (*n*, %)	33 (34.7%)	62 (37.1%)	0.704	0.90 (0.53–1.53)
Social exclusion (*n*, %)	24 (25.3%)	39 (23.4%)	0.761	1.10 (0.61–1.98)

*P-value adjusted by age and education levels.

**Male vs. female. Bold values indicate the significance.

### Multiple mediation analysis for school bullying quantity

**Figure 1:**
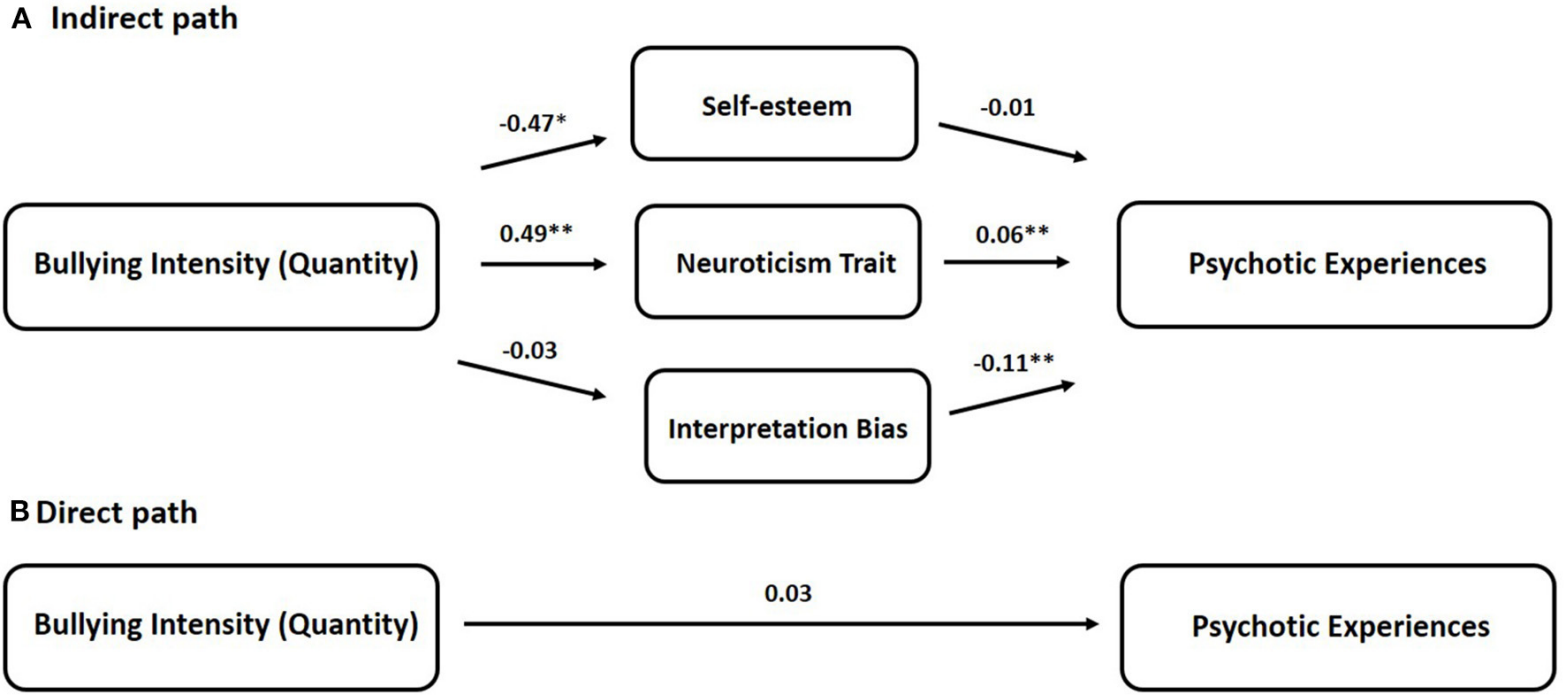
The multiple mediator model for bullying intensity (Quantity) on psychotic experiences. **(A)** Indirect path and **(B)** direct path. Unstandardized coefficient (*b*) presenting the effect of school bullying on mediators and scores of Psychotic Experiences (PE). Significant effect was found for the total effect of bullying quantity on PE scores (*p* = 0.0001), and borderline significant effect was found for the direct effect of bullying intensity (quantity) on PE scores (*p* = 0.058). The multiple-mediator model accounting for 15.96% of the variance in PE scores [*F*_(4, 257)_ = 12.04, *p* < 0.001]. *Significant at *p* < 0.05, **Significant at *p* < 0.01.

In Chinese adolescents and young adults, the quantity of bullying was negatively related to self-esteem (*b* = −0.47, *p* = 0.024) and positively related to personality traits in the neuroticism dimension (*b* = 0.49, *p* < 0.001). No significant association was detected between bullying quantity and interpretation bias. Of interest, the neuroticism trait would positively predict scores of psychotic experiences (*b* = 0.06, *p* < 0.001), and interpretation bias (positive bias) would negatively predict scores of psychotic experiences (*b* = −0.11, *p* = 0.009) (Figure 1). In the multiple mediation model, significant total effect (direct path + indirect path *via* 3 mediators) (*b* = 0.07, *p* < 0.001) and borderline significant direct effect (direct path) (*b* = 0.03, *p* = 0.058) on scores of psychotic experiences were found for bullying quantity after adjustment for age, gender, and education levels. Therefore, participants who suffered from more secondary school bullying had higher psychotic experiences later on. When taken as a set, the multiple mediation model accounted for 15.96% of the variance in scores of psychotic experiences [*F*_(4, 257)_ = 12.04, *p* < 0.001]. Specifically, the significant effect of bullying quantity on psychotic experiences was partially mediated through neuroticism trait (95% CI [0.0132, 0.0472]), but not self-esteem (95% CI [−0.0005, 0.0122]) and interpretation bias (95% CI [−0.0009, 0.0131]) (Table 3). Approximately 40.79% of the total effect was accounted for by the neuroticism trait and, to a lesser extent, interpretation bias (5.20%) and self-esteem (5.06%) (Table 3).

**Table 3:** Indirect effect of school bullying intensity (quantity) on psychotic experiences.

**Pathway**	**Effect**	**Boot SE**	**BC 95% CI**	
	***b***		**Lower**	**Upper**
Self-esteem	0.0035	0.0031	−0.0005	0.0122
Neuroticism trait	0.0278	0.0086	**0.0132**	**0.0472**
Interpretation bias	0.0035	0.0033	−0.0009	0.0131
Total	0.0348	0.0098	**0.0172**	**0.0557**
**Ratio (%)**
Self-esteem	0.0506	0.0498	−0.0082	0.1929
Neuroticism trait	0.4079	0.1579	**0.1879**	**0.7884**
Interpretation bias	0.0520	0.0565	−0.0144	0.2192
Total	0.5105	0.1873	**0.2511**	**0.9508**

b, unstandardized coefficient; SE, standard error; BC, bias corrected; CI, confidence interval; Ratio, indirect effect/total effect. Bold values indicate the significance.

### Multiple mediation analysis for school bullying duration

**Figure 2:**
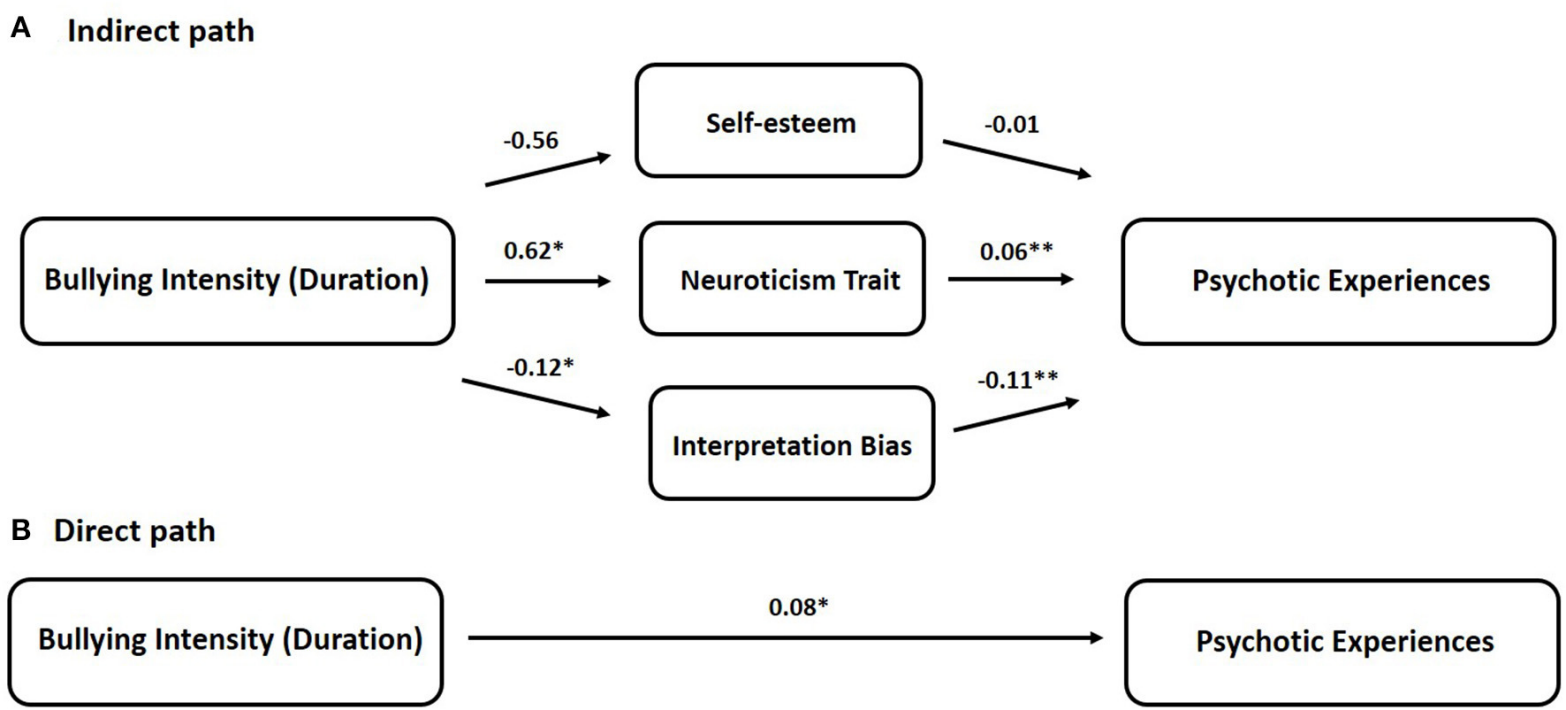
The multiple mediator model for bullying intensity (Duration) on psychotic experiences. **(A)** Indirect path and **(B)** direct path. Unstandardized coefficient (*b*) presenting the effect of school bullying on mediators and Psychotic experiences (PE). Significant effects were found for the total effect (*p* = 0.0001) and direct effect (*p* = 0.016) of bullying intensity (duration) on PE scores. The multiple-mediator model accounting for 16.47% of the variance in PE scores [*F*_(4, 257)_ = 9.38, *p* < 0.001]. *Significant at *p* < 0.05, **Significant at *p* < 0.01.

The duration of bullying was positively related to the neuroticism trait (*b* = 0.62, *p* = 0.015), and negatively related to interpretation bias (positive bias) (*b* = −0.12, *p* = 0.005). While no significant association was found between bullying duration and self-esteem (Figure 2). The significant effects of bullying duration on scores of psychotic experiences were shown for both total effect (direct path + indirect path *via* 3 mediators) (*b* = 0.13, *p* < 0.001) and direct effect (direct path) (*b* = 0.08, *p* = 0.016) after adjustment for age, gender, and education levels. The overall multiple mediation model accounted for 16.47% of the variance in scores of psychotic experiences [*F*_(4, 257)_ = 9.38, *p* < 0.001]. Different from bullying quantity, the significant effect of bullying duration on psychotic experiences was partially through two mediators, neuroticism trait (95% CI [0.0085, 0.0704]) and interpretation bias (95% CI [0.0019, 0.0295]), but not through self-esteem (95% CI [−0.0012, 0.0200]) (Table 4). Furthermore, the association effect between bullying duration and psychotic experiences was mainly attributed to the neuroticism dimension trait (27.16%), and to a lesser degree, interpretation bias (8.83%) and self-esteem (3.22%) (Table 4).

**Table 4:** Indirect effect of school bullying intensity (duration) on psychotic experiences.

**Pathway**	**Effect**	**Boot SE**	**BC 95% CI**	
	***b***		**Lower**	**Upper**
Self-esteem	0.0042	0.0049	−0.0012	0.0200
Neuroticism trait	0.0350	0.0155	**0.0085**	**0.0704**
Interpretation bias	0.0114	0.0066	**0.0019**	**0.0295**
Total	0.0505	0.0183	**0.0170**	**0.0903**
**Ratio (%)**
Self-esteem	0.0322	0.0413	−0.0120	0.1620
Neuroticism trait	0.2716	0.1329	**0.0742**	**0.6003**
Interpretation bias	0.0883	0.0682	**0.0112**	**0.2782**
Total	0.3920	0.1672	**0.1448**	**0.7910**

b, unstandardized coefficient; SE, standard error; BC, bias corrected; CI, confidence interval; Ratio, indirect effect/total effect. Bold values indicate the significance.

## Discussion

While school bullying has been regarded as a universal phenomenon, cultural variations have been implied in its prevalence and relation to psychosis disorders. Most previous studies, however, are conducted in Western populations. The current study aimed to extend the investigation to Chinese adolescents and young adults in a subclinical population that shares risk factors and neuropsychological correlations with clinical psychosis (54–56). Using a community sample from Hong Kong, we found significant psychological and cognitive pathways linking bullying quantity and duration to psychotic experiences. These findings are consistent with previous cross-sectional (57, 58) and longitudinal studies (59), which suggest important mediating roles of psychological and cognitive factors underlying the mechanism of school bullying-induced psychotic experiences.

We found that previous experiences of secondary school bullying measured by parameters of quantity and duration are associated with participants' current psychotic experiences. In line with findings in the Western population (15–19, 21), school bullying experiences can significantly predict the symptoms of psychotic experiences in Chinese adolescents and young adults. This finding implies that exposure to adverse events at secondary school is a critical risk factor for experiencing psychotic phenomena and possibly developing a psychotic disorder later. Since exposure to one type of school bullying tends to increase the risk of exposure to other types, the dose-effect of different bullying subtypes (bullying quantity) on psychotic experiences is of particular research interest and importance. As indicated by our findings, multiple bullying experiences by peers (exposure to more than one subtypes of bullying experiences) are common phenomena in secondary schools in Hong Kong (61% based on 141 bullied participants, 32.8% based on 262 total participants), and they are associated with more symptoms of psychotic experiences in our sample. Although we used self-reported retrospective questionnaires to measure secondary school bullying experiences by peers in the current study, evidence has suggested the stability of retrospective questionnaires over time in psychotic populations (60). As exposure to various bullying subtypes would lead to an accumulation of risk effects as revealed by the current study, the significant association between bullying quantity and psychotic experiences suggests that the subgroup of Chinese adolescents and young adults with multiple bullying experiences may need more care in terms of social support and targeting interventions.

Regarding the underlying mechanism, the combined indirect effect through the three mediators suggests partial mediations (point estimate of 0.0348 in the model of bullying quantity; 0.0505 in the model of bullying duration, both of which are different from zero). Subsequent investigation of the specific indirect effect demonstrates that the neuroticism trait is a significant mediator of the effect of secondary school bullying on psychotic experiences, which is supportive of our hypothesis. In the bullying quantity model, participants who suffered from more secondary school bullying subtypes had higher neuroticism traits (lower stability), and more psychotic experiences (Figure 1). Similarly, in the bullying duration model, participants with longer secondary school bullying duration had higher neuroticism traits, which were related to more psychotic experiences (Figure 2). The similar mediating patterns among the two bullying models extend our previous understanding of the importance of personality traits in the psychopathology of psychotic disorders (32). The neuroticism trait characterized by emotional instability and mood swings has been continuously related to negative affect. Participants who are high in this trait tend to suffer from more fear, worry, anxiety, and loneliness and respond worse to environmental stress. Because of its positive correlation with perceived stressors, the neuroticism trait has been used as a proxy measure of environmental stress sensitivity (61). While this trait is relatively stable due to the genetic component, evidence suggests it is still changeable over the individual life span attributed to negative environmental factors, especially adverse life events (62–64). Consistent with Wang's finding suggesting that bullying victimization is an antecedent of maladaptive personality traits (65), in the current study, exposure to bullying victimization (negative environmental factors) at secondary school would predict personality traits in the neuroticism dimension. Subsequently, the personality traits in the neuroticism dimension would further predict psychotic experiences in Chinese adolescents and young adults, which is in line with other findings in the Western context (32). Therefore, it is assumed that in the adverse environment of school bullying, victims with higher neuroticism traits are assumed to have increased sensitivity and a lower activation threshold in response to stress, which may ultimately induce psychotic experiences related to psychopathology. However, this assumed underlying mechanism needs to be further validated using prospective longitudinal studies and multiple neuroticism evaluation scales in the future.

In addition to neuroticism traits, interpretation bias appears to be another significant mediator in the bullying duration model. When looking at the directions of the mediation effect, in line with our hypothesis, we found that longer bullying duration was associated with more psychotic experiences *via* less positive but more negative interpretation bias (Figure 2). This interesting finding may be explained by the deficit cognitive model, which is suggested to be involved in developing psychotic ideation and the onset of psychotic disorder (66, 67). According to the model, adverse experiences, such as secondary school bullying, in early life, if severe or long enough, will have a negative impact on schemas of self and the around world by influencing information processing, including screening, encoding, and retrieval, which subsequently facilitate external attribution and explanatory bias. The dysfunctional cognitive beliefs will, in turn, result in the victims' social threat anticipations and thus forming paranoid delusions. This speculative cognitive model has been further indicated by evidence from neurobiological research in the striatal dopamine system (68, 69). The significant contribution of interpretation bias in the school bullying and psychotic experiences association, as shown in the current study, offers additional evidence to the deficit cognitive model, and has clinical intervention implications. Studies have recently focused on using Cognitive Bias Modification Therapy, a novel psychological intervention, to manipulate negative interpretation bias to a positive one in patients with paranoid symptoms (70). Findings from the preliminary data have shown its acceptability in the target patients (71). Suppose this psychological intervention can be further modified to fit the school environment and target bullying victims. In that case, it is expected that the early prevention of psychopathology will be achieved by altering the existing negative cognitive schema of victims and enabling them to cope with their bullying experiences in a more positive way.

There are several limitations of the current study. First, although the association between school bullying and psychotic experiences is strong in our study, we cannot draw a causal conclusion due to the cross-sectional design. Secondly, in addition to the computer-based task, the other measurements in the current study are based on self-report questionnaires, which can be influenced by memory, honesty, context, and socially expected responses. These could lead to incorrect responses or under-responses, especially for secondary school bullying experiences. Future studies are required to follow up our current findings in a longitudinal design and complement the present measurements with structured clinical interviews in a larger sample. Combining subjective and objective data collection methods should afford more thorough information regarding the issues. Moreover, there is a lack of corroborating data, such as teacher and school reports or other informants (i.e., parent/guardian) reports, in the current study, which may lead to potential information bias. Other important information of the participants (i.e., socioeconomic status and familial features) which would introduce significant confounding effects to the results, were absent from the current study. This should be improved with a better design in the future. Finally, a more comprehensive investigation on the impact of secondary school bullying on psychotic experiences, like the overlay effects of school bullying and other adverse life events, for example, domestic violence, needs future research efforts to explore in Chinese adolescents and young adults.

In the current study, we have constructed multiple mediation models to systematically investigate the mechanisms of secondary school bullying-induced psychotic experiences in a subclinical population, which has been widely used to study the psychotic disorders. For the first time, in Chinese adolescents and young adults, we identify the intermediary roles of neuroticism trait and interpretation bias, both of which underpin the linking pathways of school bullying and psychotic experiences. Our findings will facilitate the development of school-based intervention programs designed explicitly for bullied students in order to achieve early prevention of psychotic disorders.

## Data availability statement

The raw data supporting the conclusions of this article will be made available by the authors, without undue reservation.

## Ethics statement

The studies involving human participants were reviewed and approved by the Human Research Ethics Committee (HREC) of the University of Hong Kong. Written informed consent to participate in this study was provided by the participants' legal guardian/next of kin.

## Author contributions

TT conceived and designed the experiments and offered a critical revision of its content. LHC implemented the study, collected data, made the statistical analyses, and wrote the draft of the paper. All authors contributed to the article and approved the submitted version.

## Funding

This work was supported by the 28th Round Postdoctoral Fellowship/Research Assistant Professor Scheme to LHC, awarded to TT by the University of Hong Kong, and by the Small Project Funding of the University of Hong Kong Committee on Research and Conference Grants (201409176213) as well as Mental Health Research Center Mini-seed Fund of the Hong Kong Polytechinic University (P0042418) awarded to LHC.

## Conflict of interest

The authors declare that the research was conducted in the absence of any commercial or financial relationships that could be construed as a potential conflict of interest.

## Publisher's note

All claims expressed in this article are solely those of the authors and do not necessarily represent those of their affiliated organizations, or those of the publisher, the editors and the reviewers. Any product that may be evaluated in this article, or claim that may be made by its manufacturer, is not guaranteed or endorsed by the publisher.
